# Carcass Yields and Physical-Chemical Meat Quality Characteristics of Namibian Red Hartebeest (*Alcelaphus buselaphus*) as Influenced by Sex and Muscle

**DOI:** 10.3390/foods10102347

**Published:** 2021-10-01

**Authors:** Louwrens C. Hoffman, Diana L. van Schalkwyk, Magdalena Muller, Tersia Needham, Kenneth W. McMillin

**Affiliations:** 1 Department of Animal Sciences, Stellenbosch University, Private Bag X1, Matieland, Stellenbosch 7602, South Africa; foodsafetynam@gmail.com (D.L.v.S.); needham@ftz.czu.cz (T.N.); 2 Center for Nutrition and Food Sciences, Queensland Alliance for Agriculture and Food Innovation (QAAFI), The University of Queensland, Digital Agricultural Building 8115, Office 110, Gatton 4343, Australia; 3 Department of Food Science, Stellenbosch University, Private Bag X1, Matieland, Stellenbosch 7602, South Africa; mm7@sun.ac.za; 4 Department of Animal Science and Food Processing, Faculty of Tropical AgriSciences, Czech University of Life Sciences Prague, Kamýcká 961/129, 16500 Prague, Czech Republic; 5 Agricultural Center, School of Animal Sciences, Louisiana State University, Baton Rouge, LA 70803-4210, USA; kmcmillin@agcenter.lsu.edu

**Keywords:** game meat, venison, tenderness, physical quality, healthy

## Abstract

This study determined the carcass yields of red hartebeest from Namibia and compared the physical-chemical meat quality characteristics of six different muscles (*biceps femoris, infraspinatus, longissimus thoracis et lumborum, semimembranosus, semitendinosus, and supraspinatus*) for both males and females. Red hartebeest males were heavier (133.92 kg) than females (114.20 kg) but the average dressing percentage did not differ between the two sexes. Muscles from females had a lower mean shear force value of 3.59 kg/1.27 cm ø, compared to males (4.23 kg/1.27 cm ø). The most tender muscle was the *infraspinatus* of the female treatment group, while the *semimembranosus* of the male treatment group was the least tender muscle. Drip loss, cooking loss and L* (lightness) values were not affected by sex. The largest hue angle was observed in the *semitendinosus* muscle of the female treatment group (28.94°), and it was thus the lightest red muscle. The highest chroma values (17.3) were observed in the *semimembranosus* muscle. Muscle protein content averaged 20.5% over all treatment combinations, and the mean intra-muscular fat content for both male and female muscles was low (2.4%). The shoulder muscles, *infraspinatus* and *supraspinatus*, of the females had the highest fat content (2.7%). The results indicate that red hartebeest meat should be market according to specific muscles and that sex of the animals need not be considered during marketing.

## 1. Introduction

The desertification process and bush encroachment in Namibia have produced an environment that can no longer economically support increased numbers of domestic cattle. It is estimated that Namibian livestock producers lose approximately N$ 700 million (approximately USD 88.1 million) in meat production annually due to bush encroachment [1]. This phenomenon, as well as the increase in the demand for food security, spearheaded the search for other sources of high-quality proteins. 

The meat production potential of game animals from Africa has been recognized for many years [2,3,4,5,6,7,8]. Game meat is frequently a by-product of the recreational hunting industry [9]; however, the potential of harvesting game species for meat production has only been recently recognised, together with investigations into how to make it a more sustainable food source. This idea has become the latest focus worldwide [10,11,12]. Although there have been calls to remove bush/game meat from the human food chain to protect public health and biodiversity [13], it has been demonstrated that the sudden removal of this source of protein could result in the loss of food security, particularly in developing countries [10,14]. An ungulate species identified as a possible source of meat is the red hartebeest (*Alcelaphus buselaphus*) [15]. The red hartebeest is exceptionally tolerant to dry areas and poor pastures, and are found in Namibia, Botswana, Zimbabwe and South Africa (regions of the North Western Cape, Limpopo, KwaZulu-Natal, and the Free State) [16]. Previously estimated numbers of red hartebeest in the Limpopo Province were 15,000 [17], while the population density of red hartebeest in Namibia is approximately 140,000 [18,19]. In Namibia they are found on the Kalahari sands in the eastern parts of the country, and herd numbers can range from thirty to four hundred animals with an annual population growth rate of 20–32% [20]. The herd structure of the red hartebeest makes it suitable for harvesting as a group [4]. Red hartebeest are also known to roam in the same territories for long periods [21], further supporting sustainable harvest practices.

Despite their production potential, no scientific information on the physical and chemical composition of red hartebeest produced under Namibian conditions is available. When calculating the value of game animals for meat production it is important to establish the percentage of the carcass composed of edible tissues [22], while the same factors of yield, chemical composition and meat qualities are important when utilizing game meat for commercial purposes as when utilizing traditionally farmed species [23,24]. It is acknowledged that game animals show a higher yield of meat per animal when compared to domestic livestock, and eventually a greater financial return [25,26], motivating the necessity to investigate these factors in Namibian red hartebeest. 

A common consumer perception of game meat is that it is tougher than meat from domesticated livestock, although results from various studies have contradicted this [3,27,28]. Colour also plays an important role in meat quality, as it can indicate flavour, tenderness, freshness, and safety to consumers [29,30]. Game species differ in colour [30,31] compared to beef and lamb, and this can influence the purchasing behaviour of the consumer [30]. It is argued that this is due to the higher levels of myoglobin in the muscles of game species because of their greater physical activity compared to domestic livestock. Locomotive muscles are composed of different fibre types, designed for different activities, with different myoglobin content, oxygen demand, endurance capacity and lipid metabolism. Meat quality attributes, such as tenderness and colour, can thus be affected by the dominant muscle fibre type [30,32]. The most distinguishable difference between sexes is the different levels of fat deposition, which has an important impact on the game meat’s flavour [31]. Female animals tend to accumulate more fat, which was explained by Hoffman et al. [32] as the result of differences in protein assimilation efficiency and the composition of weight gain by males and females throughout their growth period. Females also mature at a more rapid rate than males and would thus be fatter at any given chronological age [33]. 

For game meat to be successfully merchandised, scientifically based information on the meat quality characteristics of red hartebeest is needed. The aims of this study were therefore to determine and compare the carcass yields of male and female red hartebeest from Namibia, and to determine and compare the physico-chemical characteristics of six different muscles (*longissimus thoracis et lumborum*, *biceps femoris*, *semimembranosus*, *semitendinosus*, *supraspinatus* and *infraspinatus*) from male and female red hartebeest.

## 2. Materials and Methods

### 2.1. Samples

Twenty-two red hartebeest (12 males and 10 females) were harvested in late summer on the experimental farm (10,300 ha) of the Neudamm Agricultural College, approximately 30 km from Windhoek, Namibia. The red hartebeest were harvested using standard techniques [4,34], killed with head or upper neck shots. After being shot, the animals were exsanguinated within 2–5 min by cutting the throat, severing the jugular veins and the carotid arteries below the jaw line. The time of death of each animal was recorded, after which each animal was issued with a unique tag number.

Animals were thereafter transported to a field abattoir, where the full body weights of the carcasses were recorded (after exsanguination). The field abattoir consisted of a hanging frame, where onto the carcasses were hoisted. Adequate lighting was provided for the cutting and evisceration processes (design and procedures followed are described by Van Schalkwyk and Hoffman [34]). Heads were labelled and removed at the junction of the atlas and axis neck *vertebrae*, using a horizontal cut to the backbone, before being weighed. The feet were removed at a point just below the *carpus* of the front legs, and the *tarsus* of the back legs.

After evisceration of the gastrointestinal tract (white offal), as well as the pluck (red offal), carcasses were hung (with the skin/hide on) from the Achilles tendon, in a refrigerated truck at a temperature ranging from 0 to 5 °C. Carcasses were then transported in the refrigerated truck to a game processing facility. At 24 h *post mortem*, the carcasses were skinned, the cold carcasses were weighed, and the dressing percentages were calculated. For the physico-chemical analyses, the *longissimus* *thoracis et lumborum* muscle was removed between the 12th and 13th ribs and the 4th and 5th *lumbar vertebrae*.

The *biceps femoris*, *semimembranosus*, *semitendinosus*, *supraspinatus* and *infraspinatus* muscles from one side of each carcass were also removed for analyses. Physical measurements were determined on fresh chilled (2–4 °C) muscle samples. Samples for chemical analyses were cut, vacuum-packed in polythene bags (80 micron thickness, water vapour transmission rate 7 cc/m^2^/day, oxygen transmission rate 50 cc/m^2^/day, carbon dioxide transmission rate 200 cc/m^2^/day, nitrogen transmission rate 12 cc/m^2^/day, Multimax, Windhoek, Namibia), labelled, and stored at −18 to −20 °C.

### 2.2. Temperature and pH 

Initial temperature and pH (pH_0_) readings at the time of death, and ultimate temperature and pH readings (pH_u_) at 24 h *post mortem*, were taken in the *M. longissimus thoracis et lumborum* of the carcasses, between the last and second-last ribs. A temperature and pH meter with automatic temperature compensation (Testo model 205, Testo AG, Lenzkirch, Germany) was used for these measurements.

### 2.3. Proximate Analyses

The moisture, protein, fat, and ash content were determined on thawed and ground muscle (±100 g) [35]. Moisture content was determined by drying the samples at 100 °C to 105 °C for 24 h (AOAC Official method 934.01) [35]. Thereafter the dried samples were placed into an oven (500 °C for 6 h) for ash determination (AOAC Official method 942.05) [35].

Protein content was determined according to AOAC official method 992.15 [35]. Dried de-fatted samples were ground with a pestle in a mortar into a fine powder. Approximately 0.15 g sample was weighed and inserted into a foil wrap designed for a Leco protein analyzer (LECO FP-528 Nitrogen Analyzer, Leco Corporation, 3000 Lakeview Avenue, St. Joseph, MO, USA). An EDTA (ethylene diamine tetra-acetic acid) calibration sample (Part number 502-092) was analyzed with each batch of samples to ensure accuracy and recovery rate. The protein content was determined as nitrogen content multiplied by a factor of 6.25.

For fat (g/100 g) determination, samples were extracted using chloroform:methanol (2:1) [36] and 5 mL of the extract was removed, transferred to a pre-weighed beaker and evaporated for 30 min on a hot plate. The fat content was calculated from the residue. 

### 2.4. Drip Loss

Drip loss was determined by suspending a freshly cut and weighed meat sample of ±60 g (ca. 15 mm-thick slice, cut perpendicular to the muscle fibre direction of the meat) in an inflated polythene bag, without the sample touching the sides of the polythene bag. The bags were left in a cold room at 1–5 °C for 24 h before the samples were removed, blotted and reweighed. The drip loss was expressed as a percentage of the weight of the fresh sample [37]. 

### 2.5. Cooking Loss

Cooking loss was determined by placing a freshly cut and weighed meat sample of ±60 g (ca. 15 mm thickness, cut perpendicular to the muscle fibre direction of the meat) in a polythene bag. Sealed bags were cooked in a water bath at ±80 °C for 1 h. Samples were then removed from the water bath, the fluid purge drained from the bags and the samples cooled under running water, while still in bags. The remaining liquid was decanted afterwards, the samples were blotted and weighed again. The cooking loss was expressed as a percentage of the initial weight [37].

### 2.6. Physical Tenderness

Physical tenderness of the muscles was determined by measuring the Warner-Bratzler (WBS) shear force values on a Universal Testing Machine (Model 4444, Apollo Scientific, Midrand, South Africa), fitted with a Warner-Bratzler blade (1.2 mm thick with a triangular opening, 13 mm at the widest point and 15 mm high). Four cylindrical cores, with a 1.27 cm diameter, of each cooked muscle (see above) were removed with a hand-coring device. Samples were cut perpendicular to the longitudinal axis of each muscle. Maximum shear force values (kg/1.27 cm ø) of the cylindrical cores of cooked meat were measured (at a cross head speed of 33.3 mm/s) and recorded for each of the four samples. Higher values were indicative of a tougher sample [37]. 

### 2.7. Colour Measurements

Colour of the muscles was determined by using a Colour-guide 45°/0° colorimeter (CAT no: 6805; BYK-Gardner, Colombia, MD, USA) set at d:0° (diffuse illumination/0° viewing angle; specular component included) with a standard observer angle of CIE: 2°, after calibrating the instrument using the white calibration tile, as per supplier’s instructions. The freshly cut muscles were allowed to bloom for a period of 30 min before measurements were taken. Three measurements were taken at randomly selected sites on the sample surfaces. The CIE L*, a* and b* values were determined, where L* indicates lightness, a* the red-green range, and b* the blue-yellow range. The hue angles and chroma values were calculated as hue angle = tan ^−1^(b*/a*) and chroma = ((a*)^2^ + (b*)^2^)^0.5^. 

### 2.8. Statistical Analyses

The experiment followed a completely randomized design. For the carcass yield, pH and temperature measurements, the main factor was sex (male and female) and the data analysed using a ttest. The two main factors for the remaining physico-chemical analyses were sex (male and female) and muscle (*biceps femoris, infraspinatus, longissimus dorsi (thoracis et lumborum)*, *semimembranosus*, *semitendinosus*, *supraspinatus*). The experimental unit was a single carcass. Data were analysed by general linear models, using a model considering the effects of sex and muscle and their interaction as fixed effects, fitting carcass weight as a covariate in the linear model. Shapiro–Wilk tests were used to test for deviation from normality [38]. Following confirmation, data were analysed by general linear models, using a model considering the effects of sex and muscle and their interaction as fixed effects, fitting carcass weight as a covariate in the linear model. The general linear model’s procedure (PROC GLM) of SAS software (Version 9.4; SAS Institute Inc., Cary, NC, USA). was used in analyses of variance of the various response variables, and the least squares means were obtained for the main and interaction effects. 

## 3. Results and Discussion

### 3.1. Carcass Yields

The mean live weights (kg), carcass weights (kg), and dressing percentages (%) of the red hartebeest are depicted in Table 1. Live weight and carcass weight differed for the main effect of sex (*p* = 0.013) with males weighing on average 133.92 kg, and females averaged 114.20 kg. Males had a higher mean carcass weight of 77.71 kg, and females had a mean carcass weight of 66.68 kg. In a similar study, the mean live body weight of male hartebeest in South Africa was observed to be 126.36 kg with a carcass weight of 68.46 kg, while females had a mean live body weight of 99.31 kg and a carcass weight of 51.48 kg [15]. Von la Chevallerie reported a live weight of 137.10 kg for male hartebeest and 126.2 kg for female hartebeest in South Africa [23]. The same study reported 142.5 kg for the live weight of male hartebeest and 126.2 kg for females in Tanzania. Ledger et al. reported carcass weights of hartebeest males of 81.50 kg and 73.20 kg for females [2]. A carcass weight of 60 kg (2-year-old) and a dressing percentage of 54% were reported for hartebeest in Kenya [31]. 

Dressing percentage (58.4%) was not influenced by the main effect of sex (*p* = 0.543). Ledger et al. reported a dressing percentage of 57.2% for male hartebeest, and 58.1% for female hartebeest [2], which corresponds with the findings in the present study. Both the male and female skin/hide contributed 7% to their live weight. Heads from male hartebeest weighed on average 10.61 kg and those of females 8.09 kg—the differences being contributed to the heavier horns of the males (both sexes have horns). 

### 3.2. Physico-Chemical Analyses

The initial temperature (Temperature_0_) measurement at the time of shooting differed (Table 2) for the main effect of sex (*p* = 0.027). This can possibly be ascribed to the fact that temperature measurements were taken between 1 and 2 h after shooting, and some carcasses were already in a more advanced state of cooling. Some of the red hartebeest were shot in terrain, which was difficult to access, which affected the initial temperature measurements. No differences were observed for sex (*p* = 0.116) for the temperatures (Temperature_u_) measured at 24 h *post mortem*, averaging 9.75 °C. The high ultimate temperatures were most probably due to the doors of the refrigerated truck being constantly opened as carcasses were loaded throughout the harvesting process.

The initial pH (pH_0_) measured varied between 6.3 and 6.4, and declined to an ultimate pH (pH_u_) of 5.7. No differences were observed for sex for both pH_0_ (*p* = 0.539) and pH_u_ (*p* = 0.621). This agrees with the findings of Onyango et al. who reported average pH values of 5.7 and 5.6 for hartebeest loins and legs, respectively [31]. The extent, and to a lesser degree the rate, of pH decline *post mortem* is a generally accepted measure of pre-slaughter stress, which can influence the quality characteristics of meat [39,40]. 

The amount of glycogen available in the muscle for *post mortem* glycolysis is fundamental for the conversion of muscle into meat and will affect the rate and extent of the pH decline, which may vary depending on the muscle, species, and nutritional status of the animal. Pre-slaughter stress is one of the factors influencing the levels of *post mortem* glycogen. In ruminants, a muscle which is deficient in glycogen (caused by pre-slaughter stress) produces dark, firm, dry (DFD) meat with high ultimate pH values [40]. The rate of pH decline is also influenced by the cooling rate of the meat [40]. However, as these carcasses were all cooled in the same environment, it can be assumed that this would have minimal influence on this parameter. Indications from the ultimate pH in this investigation are that the animals were not stressed at slaughter.

Of the various physical and chemical meat quality attributes of red hartebeest meat evaluated, it is only the shear force that showed a statistical interaction between sex and muscle; this interaction will be discussed as such whilst the influence of the main effects on the other parameters will be discussed where appropriate (Table 3). For completeness the values of the different muscle within sex are depicted in Table 3, Table 4 and Table 5. With the exception of shear force, sex had no influence on any of the physical and chemical attributes measured, whilst as expected, muscle type had a number of significant differences. 

Consumers regard tenderness as the most important eating quality attribute of meat; this attribute is most commonly measured by either a trained sensory panel or an Instron fitted with a Warner-Bratzler shear force blade (physical measurement as used in this investigation). Muscles from females had a lower mean shear force value of 3.59 kg/1.27 cm ø, compared to males, with a value of 4.23 kg/1.27 cm ø. The most tender muscle was the *infraspinatus* (IS) from the female treatment group, which did not differ from the IS of the males nor from the SS of the females whilst the SS from the males differed from the IS of the females but not the female SS. The *semimembranosus* (SM) muscles of the male treatment group had the highest (least tender) shear force values whilst the BF was the least tender muscle in the females, although the female BF did not differ from most of the other female muscles (LD, SM, ST) nor did it differ from any of the males’ muscles. This trend was also observed for springbok [41] and gemsbok [33] from Namibia, which were harvested in close proximity a few months earlier, using the same methodology of measurement. It was also observed that although there were differences in shear force values in the current study, the measured values were still low, indicating tender meat.

When seven different game species were examined for tenderness (shear force), hartebeest meat was found to be more tender (2.9 kg/cm^2^) than eland (3.37 kg/cm^2^) and gemsbok (4.09 kg/cm^2^) meat, whilst impala, wildebeest and springbok had lower shear force values of 2.75, 1.81 and 1.18 kg/cm^2^, respectively [3]. It was observed that blue wildebeest (*Connochaetus taurinus*) had shear force values of 4.91 kg/1.27 cm ø in the *longissimus dorsi* muscles [42]; the latter values were similar to that of the male animals in the current study. The texture of the muscles of the red hartebeest can be improved by utilising *post mortem* strategies, such as pelvic suspension, as has been shown in eland (*Taurotragus oryx)* [43]; although taking the size of the red hartebeest into account, this might be a challenge when the animals are harvested in challenging terrain, as sometimes found in Namibia.

Drip loss (1.1%) was not affected by sex (*p* = 0.827). The highest drip loss value was observed in the *semimembranosus* muscle of the male treatment group. Tenderness of meat is associated with cooking loss, as the ability to hold water in the meat structure is important for juiciness, and is another attribute positively correlated to tenderness. Muscles with a low cooking loss tend to be most tender, while muscles with a high cooking loss lack tenderness [44]. Cooking loss (37.1%) showed no differences (*p* = 0.463) between male and female red hartebeest muscles. Onyango reported cooking loss values of almost 29.0% for hartebeest loins and 31.0% for hartebeest legs [31]; however, the difference between these two studies could be attributed to differing methodology pertaining to the determination of cooking loss. 

The means for CIE L*, a* and b* colorimetric values, hue angle and chroma for the different muscles, as affected by sex, are represented in Table 4. No differences were observed for the L* values for the main effect of sex (*p* = 0.612) showing a mean L* (lightness) value of 33.50. Onyango reported L* values of 39.45 for hartebeest in Kenya [31]. The b* values averaged at 9.06, with no differences between sex (*p* = 0.621) groups, whilst b* values close to 6.00 were observed for hartebeest in Kenya [31]. No sex differences (*p* = 0.622) were observed for a* values, averaging 13.31. The *semimembranosus* muscle, in both males and females, had the highest a* values, and thus appeared more red than other muscles. The mean hue angle calculated was 26.74°. The largest hue angle was observed in the *semitendinosus* muscle of the female treatment group, being the lightest red muscle. No differences (*p* = 0.991) were observed for chroma values for the main effect of sex. The highest chroma values (17.3) were observed in the *semimembranosus* muscle of both the male and female groups indicating higher saturation. Game meat has characteristic colour measurement properties, with typical values of L* = 33.08, a* = 13.60, b* = 10.29, chroma = 17.10 and hue = 36.85, as measured in black wildebeest muscles [40]; the hartebeest from this investigation had colour ordinates typical of game meat [30,40].

The mean values for moisture, protein, fat and ash contents for the different muscles by sex are represented in Table 5. Moisture values did not differ (*p* = 0.930) for the main effect of sex and showed a mean value of 76.1%. This is in line with the reported moisture values of 75.0% for red hartebeest in South Africa [15]. No differences were observed for the main effect of sex for protein content values (*p* = 0.249), with an average protein value of 20.5%; slightly higher protein values for male (23.3%) and female (23.1%) red hartebeest were reported in South Africa [15]. The highest protein value in the current study was observed in the *longissimus dorsi* muscle of the male group.

Fat values were not affected by sex as a main effect (*p* = 0.677). The mean intra-muscular fat value for males and females was 2.4%; a mean fat value of 2.8% for female hartebeest and 4.8% for male hartebeest were reported in South Africa [15]. Interestingly, lower fat values of 1.8% for hartebeest legs and 1.0% for hartebeest loins were reported in Kenya [31]. In the present study, the shoulder muscles (*infraspinatus* and *supraspinatus*) of the female treatment group were observed to have the highest fat value (2.7%) of the different muscles investigated. Although there were slight differences between sex and muscles for the ash contents (Table 5), these were negligible. A mean ash value of 1.2% was observed for the pooled data from male and female hartebeest muscles. 

## 4. Conclusions

This study investigated the carcass yields and quality characteristics of various commercially important muscles from both male and female red hartebeest from Namibia. Although the live weight of red hartebeest in Namibia was lower than observed in previous studies for this species in South Africa and Tanzania, the mean dressing percentages were higher than that of previous studies. Furthermore, no significant differences were observed between males and females for dressing percentage. The *M. infraspinatus* of the female group was observed to be relatively more tender than other muscles. Overall, female animals had meat with lower shear force values compared to that from male animals. Muscles could be grouped in terms of tenderness, i.e., based on shear force values and other physical and chemical composition; as the meat quality and nutritional profile of the muscles only differed slightly between sexes, the meat of red hartebeest would not need to be marketed separately based on sex. However, the meat industry could consider marketing individual muscles for specific markets, and further research on the relative nutritional value and sensory properties of this species is required for cut-specific marketing. The low total fat of the muscles is also noteworthy, and it would be interesting to see what the fatty acid composition of the meat is, not only from a human nutrition perspective but also from a fresh meat shelf-life stability perspective. 

## Figures and Tables

**Table 1:** Mean carcass yields (±standard errors) and least significant differences (LSD) for male and female red hartebeest from Namibia.

	Live Weight (kg)	Carcass Weight (kg)	Dressing Percentage (%)
Male	133.92 ± 4.910	77.71 ± 3.059	58.0 ± 0.66
Female	114.2 ± 5.221	66.68 ± 1.935	58.9 ± 1.36
*p*-value	0.013	0.013	0.543

**Table 2:** Mean values (±standard errors) and least significant differences (LSD) for pH and temperature for male and female red hartebeest from Namibia.

	pH_0_	pH_u_	Temperature_0_ (°C)	Temperature_u_ (°C)
Male	6.3 ± 0.10	5.7 ± 0.04	23.28 ± 1.185	10.40 ± 0.544
Female	6.4 ± 0.13	5.7 ± 0.06	28.09 ± 1.639	8.97 ± 0.696
*p*-value	0.539	0.621	0.027	0.116

pH_0_ and Temperature_0_ = initial pH and temperature; pH_u_ and Temperature_u_ = ultimate pH and temperature.

**Table 3:** Mean values (±standard errors) and least significant differences (LSD) for shear force, drip loss and cooking loss for the *longissimus dorsi* (LD), *biceps femoris* (BF), *semimembranosus* (SM), *semitendinosus* (ST), *supraspinatus* (SS) and *infraspinatus* (IS) muscles from male and female red hartebeest from Namibia.

	Shear Force (kg/1.27 cm ø)	Drip Loss (%)	Cooking Loss (%)
**Male (*n* = 12)**			
BF	4.2 ^bc^ ± 0.320	1.2 ± 0.13	37.9 ^abc^ ± 0.39
IS	3.42 ^cde^ ± 0.159	1.0 ± 0.06	32.9 ^f^ ± 0.56
LD	4.78 ^ab^ ± 0.408	1.2 ± 0.20	35.8 ^de^ ± 0.69
SM	5.43 ^a^ ± 0.413	1.4 ± 0.24	39.5 ^ab^ ± 0.52
ST	3.76 ^cd^ ± 0.229	1.0 ± 0.08	39.3 ^ab^ ± 0.83
SS	3.76 ^cd^ ± 0.122	1.0 ± 0.05	38.9 ^abc^ ± 0.35
**Female (*n* = 10)**			
BF	4.08 ^bc^ ± 0.346	1.3 ± 0.29	37.1 ^cd^ ± 0.78
IS	2.80 ^e^ ± 0.147	0.9 ± 0.11	33.9 ^ef^ ± 0.89
LD	3.82 ^c^ ± 0.329	1.2 ± 0.13	33.9 ^ef^ ± 0.95
SM	3.94 ^c^ ± 0.284	1.2 ± 0.20	38.5 ^abc^ ± 0.62
ST	3.92 ^c^ ± 0.231	1.1 ± 0.12	39.9 ^a^ ± 0.84
SS	2.97 ^de^ ± 0.161	1.0 ± 0.13	37.6 ^bcd^ ± 1.30
*p*-values for for the main effects of season and muscle type, and their interaction			
**Sex**	0.001	0.827	0.463
**Muscle**	0.029	0.129	<0.001
**Interaction**	0.024	0.960	0.437

^a–f^ Values in the same column with different superscripts differ (*p* ≤ 0.05).

**Table 4:** Mean values (±standard errors) and least significant differences for CIE L*, a* and b* values, hue angle and chroma for the *longissimus dorsi* (LD), *biceps femoris* (BF), *semimembranosus* (SM), *semitendinosus* (ST), *supraspinatus* (SS) and *infraspinatus* (IS) muscle from male and female red hartebeest from Namibia.

	CIE L*	CIE a*	CIE b*	Hue Angle	Chroma
**Male (*n* = 12)**					
BF	34.19 ^abcd^ ± 0.651	13.29 ^abcd^ ± 0.376	9.45 ^ab^ ± 0.328	27.81 ^abcd^ ± 0.650	16.37 ^abc^ ± 0.447
IS	34.52 ^abc^ ± 0.841	13.20 ^abcd^ ± 0.301	8.68 ^bc^ ± 0.295	26.27 ^bcde^ ± 0.803	15.86 ^abc^ ± 0.313
LD	32.38 ^de^ ± 0.634	12.79 ^cd^ ± 0.389	7.82 ^c^ ± 0.464	24.44 ^e^ ± 0.751	15.06 ^c^ ± 0.557
SM	33.21 ^bcde^ ± 0.741	14.21 ^a^ ± 0.342	9.85 ^ab^ ± 0.494	27.10 ^abcd^ ± 0.760	17.34 ^a^ ± 0.521
ST	34.68 ^ab^ ± 1.051	13.09 ^abcd^ ± 0.408	9.47 ^ab^ ± 0.698	27.76 ^abcd^ ± 1.048	16.22 ^abc^ ± 0.712
SS	33.18 ^bcde^ ± 0.594	14.01 ^ab^ ± 0.227	9.11 ^abc^ ± 0.246	25.94 ^bcde^ ± 0.763	16.77 ^ab^ ± 0.190
**Female (*n* = 10)**					
BF	33.70 ^abcd^ ± 0.879	12.55 ^d^ ± 0.661	9.02 ^abc^ ± 0.729	28.08 ^ab^ ± 1.072	15.56 ^bc^ ± 0.839
IS	33.39 ^bcde^ ± 0.758	13.74 ^abc^ ± 0.312	8.76 ^bc^ ± 0.458	25.43 ^de^ ± 0.987	16.37 ^abc^ ± 0.422
LD	31.38 ^e^ ± 0.390	12.41 ^d^ ± 0.570	7.88 ^c^ ± 0.218	25.61 ^cde^ ± 0.700	14.86 ^c^ ± 0.550
SM	33.13 ^bdec^ ± 0.728	13.89 ^abc^ ± 0.510	10.16 ^a^ ± 0.753	27.93 ^abc^ ± 1.020	17.29 ^a^ ± 0.819
ST	35.77 ^a^ ± 0.924	13.06 ^bcd^ ± 0.497	9.84 ^ab^ ± 0.517	28.94 ^a^ ± 0.613	16.38 ^abc^ ± 0.679
SS	32.47 ^cde^ ± 0.728	13.37 ^abcd^ ± 0.286	8.66 ^bc^ ± 0.396	25.85 ^bcde^ ± 1.175	16.00 ^abc^ ± 0.272
*p*-values for for the main effects of season and muscle type, and their interaction					
**Sex**	0.612	0.622	0.621	0.407	0.991
**Muscle**	0.063	0.031	0.207	0.038	0.184
**Interaction**	0.700	0.623	0.844	0.828	0.747

^a–e^ Values in the same subgroup of variables with different superscripts differ (*p* ≤ 0.05).

**Table 5:** Mean values (±standard errors) and least significant differences (LSD) for moisture, protein, fat and ash values for the *longissimus dorsi* (LD), *biceps femoris* (BF), *semimembranosus* (SM), *semitendinosus* (ST), *supraspinatus* (SS) and the *infraspinatus* (IS) muscles from male and female red hartebeest from Namibia.

	Moisture (%)	Protein (%)	Fat (%)	Ash (%)
**Male (*n* = 12)**				
BF	76.5 ^ab^ ± 0.37	20.4 ^cd^ ± 0.19	2.2 ± 0.26	1.2 ^a^ ± 0.07
IS	76.8 ^a^ ± 0.20	20.0 ^cde^ ± 0.34	2.5 ± 0.32	1.1 ^ba^ ± 0.02
LD	74.9 ^d^ ± 0.36	21.8 ^a^ ± 0.47	2.3 ± 0.33	1.2 ^ba^ ± 0.07
SM	75.8 ^bcd^ ± 0.42	21.0 ^abc^ ± 0.51	2.3 ± 0.30	1.2 ^ba^ ± 0.06
ST	76.1 ^abc^ ± 0.30	20.7 ^bcd^ ± 0.45	2.6 ± 0.27	1.2 ^a^ ± 0.04
SS	76.7 ^ab^ ± 0.29	19.8 ^de^ ± 0.41	2.6 ± 0.35	1.1 ^ab^ ± 0.07
**Female (*n* = 10)**				
BF	76.4 ^abc^ ± 0.55	20.3 ^cde^ ± 0.61	2.3 ± 0.23	1.2 ^ab^ ± 0.07
IS	76.9 ^a^ ± 0.39	19.3 ^e^ ± 0.35	2.7 ± 0.16	1.0 ^b^ ± 0.07
LD	75.5 ^cd^ ± 0.44	21.5 ^ab^ ± 0.45	2.1 ± 0.22	1.2 ^ab^ ± 0.07
SM	75.8 ^bcd^ ± 0.52	20.8 ^abcd^ ± 0.43	2.3 ± 0.25	1.2 ^ab^ ± 0.08
ST	76.0 ^abc^ ± 0.27	20.6 ^bcd^ ± 0.38	2.4 ± 0.27	1.3 ^a^ ± 0.06
SS	76.3 ^abc^ ± 0.33	19.9 ^cde^ ± 0.39	2.7 ± 0.21	1.1 ^ab^ ± 0.07
*p*-values for for the main effects of season and muscle type, and their interaction				
**Sex**	0.941	0.249	0.677	0.639
**Muscle**	0.050	0.017	0.607	0.023
**Interaction**	0.961	0.861	0.948	0.878

^a–e^ Values in the same column with different superscripts differ (*p* = 0.05).

## Data Availability

The data presented in this study are available on request from the corresponding author.
